# Notes on Preparations for the Sick

**Published:** 1898-03

**Authors:** 


### Notes on Preparations for the Sick.

**Ammonol.**—The Ammonol Chemical Co., New York.—This is described as a stimulant analgesic and antipyretic coal-tar product. Being an ammoniated product, it has no depressing effect on the heart; and being alkaline, it is of especial value in gastralgia depending on hyper-acidity. It is said to be very efficacious in the treatment of migraine, and also gives speedy relief to dysmenorrhœa. The dose is 5 to 20 grains in powder or in tablets.

**Anti-Typhoid Serum.**—Burroughs, Wellcome & Co., London. —It is generally admitted that the serum diagnosis of typhoid fever has passed beyond the experimental stage, and that the Widal test gives us a means of establishing the diagnosis of typhoid on more trustworthy grounds than heretofore, but whether as much as this can be said of the therapeutic use of an anti-typhoid serum is very doubtful. The serum has been found to have an indisputable bactericidal action, and has been thought to influence beneficially animals infected with virulent cultures of typhoid bacilli. Direct clinical evidence in man is scanty; doses of 10 to 20 c.c. may be injected without serious consequences, and also without any indication of improvement.

It is clear that the serum should be given in the early stages if it is to be of any use: it can be of little value after the fourteenth day, and at this stage we are reluctant to interfere actively by injection for mere experimental purposes.

The serum is prepared in two forms; a fluid one is sent out in bottles of 10 c.c., and a dry one in phials containing the equivalent dose in scale form readily soluble in sterilised water.

In two cases in which we used it, it was not easy to say whether any definite result ensued: one case did well after five injections, amounting to a total of 11 c.c., extending from the fifteenth to the twenty-third day: the temperature fell on the twenty-seventh day, but did not remain at the normal till after the thirty-third day. The other case was a very severe one, with hemorrhages on the tenth and eleventh days: the temperature range continued high, and on the twenty-fifth day a dose of 10 c.c. was injected: a second dose of 10 c.c. was given on the twenty-seventh day; the temperature fell somewhat after each dose, but the patient died on the twenty-eighth day. We are not of opinion that these two cases give evidence against the use of the serum; neither do they give us much encouragement to expect benefit from its use.

---

**Cream of Malt with Cod Liver Oil. Cream of Malt with Cod Liver Oil and Hypophosphites. Bipalatinoids: Ferri Hypophos. Co.**—OPPENHEIMER, SON & CO., London.—The Oppenheimer Cream of Malt and its combinations are too well known to require commendation, and the bipalatinoids have all the advantages of the syrup in a very convenient form.

---

**Vin Cocakola.**—MAX MORELLA & CO., Nottingham.—This is a very pleasant fluid with no disagreeable qualities. The addition of the kola element must increase its nerve stimulation powers, and give it advantages over the ordinary preparations of coca; but we think that these medicinal wines should be looked upon as drugs and not taken as ordinary beverages in health.

---

**Salutaris Water.**—SALUTARIS WATER CO., London.—The importance to a community of a pure water supply cannot be over estimated. Even if the source be above suspicion, the risks of contamination *en route* and under ordinary domestic conditions are by no means small. To obviate these the Salutaris Company supply a distilled and aerated water which they claim to be absolutely free from unhealthy or unwelcome additions. We can speak highly of the samples we have received, and have enjoyed them much when used instead of the ordinary aerated water; but we doubt if their cost will permit of their being employed to a large extent for general table and toilet purposes, as the Company seem to hope.

**Californian Wine: No. 8 Hock.**—GRIERSON, OLDHAM & Co., London.—The notice which, in December, 1894, we gave to the wines of the Big Tree Brand did not include this variety of hock, which has since been sent to us. It can be recommended to those invalids who want something of the sort to help a poor appetite.

---

**Soloids: Zinc and Tannin Compound; Sodium Biborate Compound; Alum Compound; Eucaine Hydrochloride.**—BURROUGHS, WELLCOME & Co., London.—Those practitioners who are in the habit of ordering vaginal injections will be glad to know of the first three of these Soloids, which are convenient for readily preparing them. The first contains Zinci Sulph. gr. v., Plumbi Acet. gr. x., Ext. Op. gr. ij., Acid. Tannic. gr. j.; the second has Sod. Bibor. gr. xx. and Tinct. Op. m. x.; the third is composed of Zinci Sulph. gr. xv. and Pulv. Alum. gr. xv.

**Eucaine** is now well known as a local anæsthetic similar to cocaine, but without its toxic properties. We greatly prefer it to cocaine for most purposes, and the soloids, each containing either one or five grains, form a convenient method of using it.

